# Exposure to glyphosate-based herbicide during early stages of development increases insulin sensitivity and causes liver inflammation in adult mice offspring

**DOI:** 10.31744/einstein_journal/2022AO6778

**Published:** 2022-06-01

**Authors:** Ellen Carolina Zawoski Gomes, Jakeline Liara Teleken, Rodrigo Vargas, Ana Claudia Paiva Alegre-Maller, João Paulo de Arruda Amorim, Maria Lúcia Bonfleur, Sandra Lucinei Balbo

**Affiliations:** 1 Universidade Estadual do Oeste do Paraná Cascavel PR Brazil Universidade Estadual do Oeste do Paraná, Cascavel, PR, Brazil.; 2 Centro Universitário Fundação Assis Gurgacz Cascavel PR Brazil Centro Universitário Fundação Assis Gurgacz, Cascavel, PR, Brazil.

**Keywords:** Glyphosate, Endocrine disruptors, Glucose metabolism disorders, Inflammation, Herbicides, Mice, Inbred C57BL

## Abstract

**Objective:**

To investigate the effect of pre and postnatal exposure to a glyphosate-based herbicide on glucose metabolism and liver histology in adult F1 mice offspring.

**Methods:**

Female mice (C57Bl/6) received 0.5% of glyphosate (Roundup Original DI^®^) in drinking water or purified water (Glyphosate Group and Control Group respectively) during pregnancy and lactation. Offspring (F1) were submitted to glucose and insulin tolerance tests and euthanized on postnatal day 150. Body and plasma parameters, and liver histology were analyzed.

**Results:**

Exposure to glyphosate reduced maternal body weight gain during pregnancy and lactation, with no impacts on litter size. Pre and postnatal exposure to glyphosate did not affect body parameters but increased glucose tolerance on postnatal day 60. In spite of glucose tolerance normalization by postnatal day 143, this effect was associated with higher insulin sensitivity relative to mice in the Control-F1 Group. Mice in the Glyphosate-F1 Group had mild and moderate lobular inflammation in the liver.

**Conclusion:**

Maternal exposure to glyphosate affected insulin sensitivity and caused hepatic inflammation in adult F1 mice offspring.

## INTRODUCTION

Glyphosate (N-(phosphonomethyl) glycine) is a broad-spectrum, non-selective, systemic organophosphate herbicide.^(1)^ Indiscriminate use of this herbicide may harm the environment as well as animal and human health^(2)^ due to increased exposure via contaminated soil, water and food.^(3)^ Glyphosate-based herbicides (GBH) are the most commonly used pesticides worldwide. Herbicides such as GBH account for approximately 45% of pesticides used in Brazilian agriculture.^(4)^ Use of GBH has increased after the development of genetically-modified species resistant to glyphosate. These herbicides can be used at any stage of plant development.^(5)^

The effects of glyphosate on human health have been associated with its identification as a potential endocrine disruptor chemical (EDC). Endocrine disruptor chemicals are exogenous substances capable of interfering directly with the endocrine system. These chemicals mimic the action of natural hormones, causing adverse effects on living organisms and/or their progeny.^(3,6,7)^ Glyphosate-based herbicide formulations are thought to act as EDCs in the adrenal, thyroid and reproductive systems.^(8-10)^ Currently, much attention is being given to exposure to EDCs during critical periods of development, such as gestation and lactation, during which these substances may impact the developmental trajectory of the fetus, causing permanent morphofunctional changes in tissues and/or organs, with or without growth disturbances. Such changes may lead to “programming”, a term used to define the process through which insults or stimuli during a critical period of fetal development elicit changes in the course of life, with irreversible consequences.^(11)^

Systems involved in maintenance of glucose and lipid homeostasis are susceptible to reprogramming during stages of tissue differentiation and organ development, which begin in fetal life and continue throughout childhood. In human beings, pancreatic islets and liver development start in the 3^rd^ and 12^th^ week of gestation respectively and continue until the end of lactation. In mice, similar organogenesis begins in the 8^th^ and 7^th^ days of pregnancy respectively and persists until the start of lactation. Insults during lactation may also trigger programming and amplify the effects of in-utero exposure on metabolic processes throughout life.^(12)^

*In vitro* and *in vivo* studies with rodents have shown that acute exposure to EDCs causes changes in β-pancreatic cells, affecting the regulation of insulin secretion and the action of this hormone. Adipocyte differentiation in response to EDCs has also been observed and may cause insulin resistance, a determining factor in the pathophysiology of metabolic syndrome, obesity and type 2 *diabetes mellitus*.^(13)^

Specific and direct actions of glyphosate on the reproductive, the thyroid and central nervous system have been described in exposed individuals and their offspring. However, the effects of these substances on glucose and lipid metabolism have not been widely investigated. Studies examining the direct effects of exposure to GBH formulations in a fish species have shown that liver, muscle and plasma glucose levels decline after exposure. Moreover, GBH exposure increases transaminase levels, suggesting liver cell damage.^(14)^ Few studies have examined the effects of GBH on metabolic functions in mammals. Exposure to GBH for 12 weeks increased blood glucose levels in Wistar rats.^(15)^ Sub-chronic exposure of Wistar rats to GBH induced leakage of the hepatic intracellular enzymes alanine aminotransferase and aspartate aminotransferase, suggesting irreversible damage to hepatocytes.^(16)^ Maternal exposure to glyphosate during pregnancy and lactation led to increased hepatic lipid peroxidation in mothers and particularly in their offspring. Increased levels of the hepatic enzyme glutathione peroxidase in offspring was also observed.^(17)^ A recent study revealed that glyphosate excretion is associated with steatohepatitis and advanced liver fibrosis in patients with fatty liver disease.^(18)^

This study provides the first insights into the impacts of maternal exposure to the GBH Roundup during pregnancy and lactation. Effects on body parameters, glucose tolerance, insulin sensitivity and liver histology in adult F1 mice offspring.

## OBJECTIVE

To investigate the effects of pre and postnatal exposure to glyphosate-based herbicide on glucose metabolism and liver histology in adult F1 mice offspring.

## METHODS

### Chemicals

The GBH used in this study was Roundup Original DI^®^ (Monsanto, São Paulo, SP, Brazil). This formulation contains 445g/L of N-phosphonomethylglycine diammonium salt, which corresponds to 370g/L (37.0%m/v) of the active ingredient glyphosate.

### Study period

Experiments were carried out from May 2017 to March 2018.

### Maternal groups and glyphosate exposure

Male and female C57Bl/6 mice (60-90 days old, 20-25g of body weight) were housed in polypropylene cages under controlled temperature (28±2°C) and lightning (12-hour dark/light cycles) conditions. Mice were fed with standard rodent chow diet (Supralab Medicina Diagnóstica, Itaboraí, RJ, Brazil) *ad libitum* and had free access to purified water. Experimental procedures were performed in compliance with the Ethics Committee on Animal Use (CEUA) of *Universidade Estadual do Oeste do Paraná* (UNIOESTE).

For mating purposes, two receptive female mice and one sexually-active adult male mouse were housed in a cage during the dark period (7 p.m. to 7 a.m.). On the following morning, vaginal smears were obtained from all female mice. Pregnancy was confirmed by the presence of spermatozoa in the vaginal smears or when female remained in diestrus for four days during the estral cycle.

Pregnant female mice were randomly allocated to one of two experimental groups. During pregnancy (GD4 to GD21) and lactation (30 days), females in the Glyphosate Group (GBH, n=9) received 0.5% of glyphosate (Roundup Original DI^®^, Monsanto, Brazil) via drinking water, whereas females in the Control Group (CTRL, n=11) received pure water. The 0.5% dose used was derived from previous studies carried out by Daruich et al. and Teleken et al.^(19,20)^ The estimated average daily intake was approximately 420mg/kg/day. Hence, the estimated daily dose was below the no-observed-adverse-effect level (NOAEL) of Roundup in mice (500mg/kg/day).^(21)^

Body weight, food and water intake were measured weekly. Ten days after weaning, female mice were euthanized and body parameters, fasting glucose and insulin levels verified.

### Offspring groups

Pup birth day was defined as postnatal day 0 (PND0). Offspring were weaned on PND30. Only male mice were used in this study. Offspring were designated according to maternal treatment to form two experimental groups: CTRL-F1 (n=16) and GBH-F1 (n=10), selected out of 11 and 9 litters respectively. Mice received purified water and rodent standard chow diet (Supralab Medicina Diagnóstica, Itaboraí, RJ, Brazil) *ad libitum* until PND150, when they were euthanized. Body weight and food intake was measured weekly throughout adult life (PND60 to PND150).

### Glucose tolerance test

On PND60 and PND143, male F1 mice were submitted to the oral glucose tolerance test (OGTT). After 8 hours of fasting, blood was collected from the tail for fasting glucose measurement (time 0) using a glucometer and test strips (G-Tech Free^®^, SD Biosensor, Korea). Mice were then fed oral glucose solution (1.5g/kg) and glucose measurements repeated at 15, 30, 60, 90 and 120 minutes. For statistical analysis, values were normalized and the delta value (Δ) obtained using the following formula:


Δ=T×100/T0−100

### Insulin tolerance test

On PND145, male F1 mice were submitted to the insulin tolerance test. After 2 hours of fasting, tail blood was collected and basal glucose measurements obtained using a glucometer and test strips (G-Tech Free^®^, SD Biosensor, Korea). Glucose measurements were then repeated within 3, 6, 9, 12, 15 and 18 minutes of intraperitoneal administration of regular insulin (0.75IU/kg). Absolute glucose values were used in statistical analysis. For K_ITT_ calculation, data were log-transformed into the natural logarithm of Y (Y=Ln(Y)) followed by a linear regression test. The slope of the glucose curves was used to examine insulin sensitivity in experimental groups.

### Euthanasia

Mothers and offspring were fasted for 8 hours and glucose levels checked (G-Tech Free^®^, SD Biosensor, Korea) prior to euthanasia. Mice were weighed and anesthetized with xylazine (9mg/kg) (Anasedan^®^, Vetbrands, Brazil) and ketamine (90mg/kg) (Dopalen^®^, Vetbrands, Brazil). Once the skin reflex was absent, the naso-anal length was measured, and blood collected into a heparinized syringe by cardiac puncture. Blood samples were transferred to a tube and centrifuged (12,600g for 10 minutes at 4°C). Plasma was stored at -80°C for subsequent insulin measurement by radioimmunoassay. Laparotomy was performed and the following organs and tissues extracted and weighed: pancreas, liver, brown and white adipose tissue, retroperitoneal and perigonadal fat, and soleus and extensor digitorum muscles.

### HOMA index

Insulin resistance and β-cell function were evaluated using the homeostasis model assessment (HOMA – IR and β respectively). Fasting insulin (*μ*U/L) and blood glucose (mmol/L) values were used for this purpose.

HOMA-IR and HOMA-β were calculated using the following formulas:


$$\begin{array}{c} \left(\text{HOMA IR}=\text{fasting insulin}\times \text{fasting glucose}\right)/22.5\\ \left(\text{HOMA}\beta =20\times \text{fasting inslulin}\right)/\left(\text{fasting glucose}-3.5\right) \end{array}$$

### Liver histology

Fragments of the right hepatic lobe were collected from F1 mice in cross-sectional direction, from the center to the margin of the organ. Liver samples were fixed in Carson formalin solution (Formaldehyde 37 wt %,10%; methanol, 1.5% and Phosphate Buffered Saline/PBS, pH 7.4, 88.5%) for 24 hours, washed in running water and dehydrated in increasing concentrations of alcohol. Samples were then diaphanized in xylol, embedded in Paraplast^®^ (Sigma Co, St Louis, MO) and cut into 5μm-thick slices using a manual rotary microtome (Olympus 4060) equipped with a steel knife. Tissue sections were stained with hematoxylin-eosin (HE) for investigation of steatosis, inflammation and hepatocellular injury. Mallory’s trichrome stain was used for collagen fiber identification. For steatosis investigation, tissue vacuolization was evaluated by field. Generalized liver inflammation was scored according to the number of leukocyte clusters per 200x field, as described by Kleiner et al.^(22)^ Images were analyzed using an optical microscope (Olympus BX61) equipped with a digital camera (Olympus DP71) and DP Controller 3.2.1.276 software.

### Data analyses and statistics

Data were expressed as means±standard error of the mean. The Shapiro-Wilk test was used for normality testing. Parametric data were analyzed using the unpaired Student’s *t*-test. Non-parametric data were analyzed using the Mann-Whitney test. The level of significance was set at p<0.05. Analyses were performed using GraphPad Prism software, version 6.0 for MAC (GraphPad Software^©^) and the statistical package R (R Coreteam, 2015).

## RESULTS

### General maternal parameters

**Figure 1:**
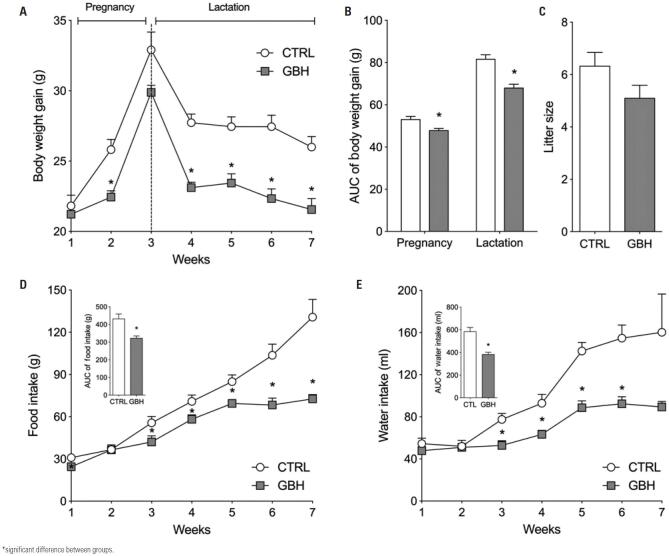
Effect of glyphosate exposure during pregnancy and lactation. (A) Body weight gain during pregnancy and lactation; (B) Area under curve of body weight gain; (C) Litter size; (D) Food and (E) Water intake during pregnancy and lactation

GBH: Glyphosate Group; CTRL: Control Group.

Glyphosate administration reduced body weight gain during pregnancy (p=0.0051) and lactation (p<0.0001) in the GBH relative to the CTRL Group (Figures 1A and B). However, the number of offspring did not differ between groups (p=0.1088; Figure 1C), as shown by Teleken et al.^(20)^ Glyphosate exposure also reduced food (p=0.0026) and water (p=0.0002) intake in females in the GBH compared to the CTRL Group (Figures 1D and E respectively).

### Pregnancy development and parental care

Glyphosate administration had negative impacts on gestation and maternal care in the GBH Group. In 43% of mothers exposed to glyphosate (0.5% in drinking water), gestation went uneventful and there were no negative impacts in parental care. However, of remaining mothers (57%), 19% had incomplete gestation and 38% did not breastfeed or killed their pups.

All other experiments in this study were carried out with offspring of the 43% of mothers who completed pregnancy and had no other adverse events.

### Maternal organ weight, glucose and insulin levels

Glyphosate exposure had no impacts on perigonadal adipose tissue, pancreas, soleus, or extensor digitorum muscle weight (Table 1). Glucose levels, insulin levels, HOMA-IR and HOMA-β indexes did not differ significantly between groups (Table 1).

**Table 1:** Maternal features and fasting plasma of dams exposed to 0.5% glyphosate during pregnancy and lactation

Parameters	CTRL	GBH	p value
Perigonadal fat (mg/mg body weight)†	0.68±0.07	0.85±0.68	0.12
Pancreas (mg/mg body weight)†	0.58±0.06	0.91±0.15	0.08
Extensor digitorum longus muscle (mg/mg body weight)*	0.03±0.002	0.04±0.007	0.06
Soleus muscle (mg/mg body weight)†	0.02±0.001	0.03±0.003	0.06
Glucose (mg/dL)†	104.9±5.83	95.44±4.99	0.24
Insulin (ng/mL)†	0.48±0.08	0.49±0.10	0.97
HOMA-IR†	3.43±0,59	2.92±1.14	0.70
HOMA-β†	79.14±21.85	93.68±30.73	0.71

Data expressed as mean±standard error of mean.

* Mann-Whitney test

† tudent’s *t* test. p value<0.05.

CTRL: Control Group; GBH: Glyphosate Group. CTRL (n=4-11) and GBH (n=3-9).

### General parameters of male F1 offspring

**Figure 2:**
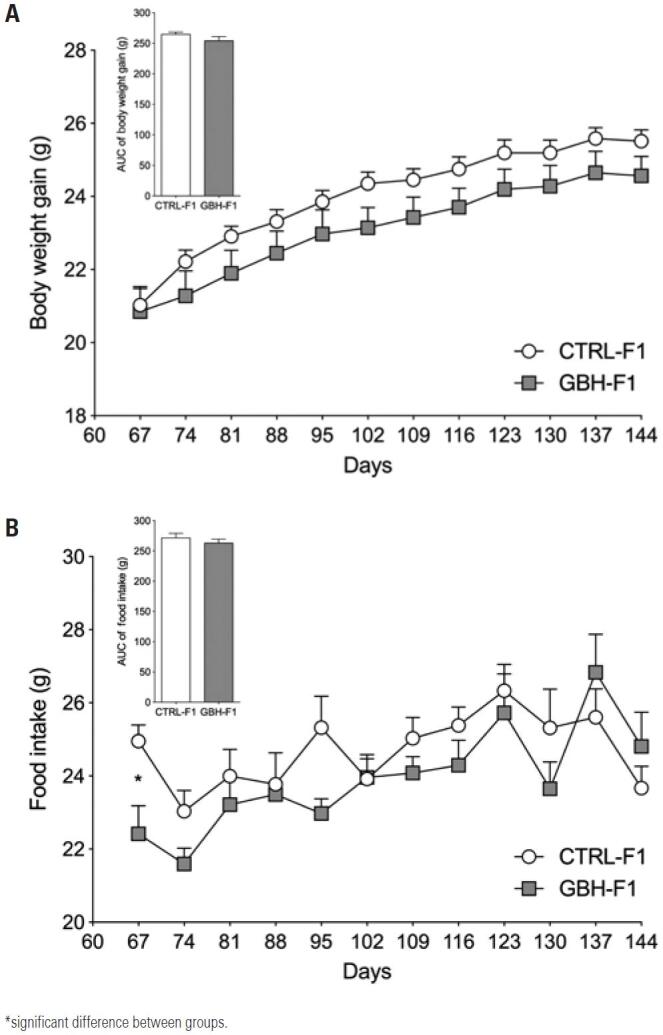
Effect of maternal exposure to glyphosate on F1 offspring. (A) Body weight gain in adult life and (B) Food intake in adult life

GBH: Glyphosate Group; CTRL: Control Group.

Maternal exposure to glyphosate did not affect body weight gain from PND60 to PND150 in the GBH-F1 Group (p=0.1222; Figure 2A). Food intake was similar in both groups (p=0.8459; Figure 2B). Likewise, pre and postnatal exposure to glyphosate did not affect naso-anal length, Lee’s index or tissue weight (retroperitoneal and perigonadal fat, brown adipose tissue, soleus and longus extensor muscle) in adult F1 offspring (Table 2).

**Table 2:** Effects of pre and postnatal exposure to glyphosate on offspring features and fasting plasma parameters in male F1 mice on postnatal day 150

Parameters	CTRL-F1	GBH-F1	p value
Body weight (g)†	25.03±0.36	23.63±0.74	0.06
Naso-anal length (cm)*	9.28±0.08	9.06±0.13	0.16
Lee Index†	315.40±3.02	316.90±4.16	0.76
Retroperitoneal fat (mg)†	49.38±4.88	53.00±9.02	0.70
Perigonadal fat (mg)†	254.70±17.25	257.20±19.57	0.93
Brown adipose tissue (mg)†	76.13±4.31	66.57±7.33	0.25
Extensor digitorum longus muscle (mg)*	10.88±1.12	10.33±0.76	0.64
Soleus muscle (mg)*	13.69±3.79	9.12±1.39	0.20
Glucose (mg/dL)†	104.80±5.12	109.40±7.60	0.61
Insulin (ng/mL)†	0.25±0.03	0.29±0.05	0.42
HOMA-IR*	1.52±0.15	1.60±0.23	0.73
HOMA-β*	85.41±18.63	69.09±16.73	0.86

Data expressed as mean±standard error of mean.

* Mann-Whitney test

† Student’s *t* test. p value<0.05.

CTRL: Control Group; GBH: Glyphosate Group. CTRL (n=16) and GBH-F1 (n=7-9).

### Glucose and insulin levels, and glucose tolerance test results in male F1 offspring

**Figure 3:**
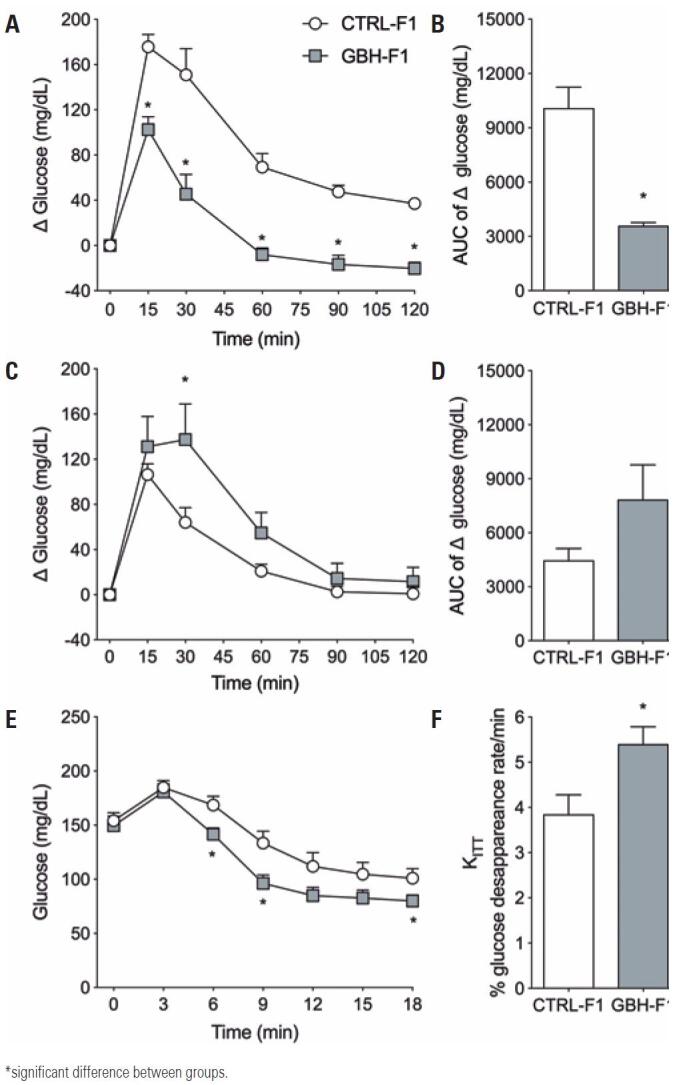
Effect of maternal exposure to glyphosate on glucose metabolism in F1 offspring. (A) Glucose tolerance test on PND60; (B) Area under curve; (C) Glucose tolerance test on PND143; (D) Area under curve; (E) Insulin tolerance test on PND145; (F) Glucose disappearance rate (KITT)

Glucose levels, insulin levels, HOMA-IR and HOMA-β indexes did not differ significantly between offspring groups (Table 2). On PND60, both groups reached maximum blood glucose levels within 15 minutes of glucose overload (1.5g/kg). Mice in the GBH-F1 Group had a 35% drop in plasma glucose levels, indicating higher sensitivity to glucose relative to mice in the CTRL-F1 Group (p=0.0028; Figure 3A and B). On PND 143, glucose tolerance did not differ significantly between groups (p=0.834; Figures 3C and D). However, glucose level decline following regular insulin administration (0.75IU/kg) was significantly higher in the GBH-F1 than in the CTRL-F1 Group (p=0.0234) (Figures 3E and F), suggesting higher sensitivity to this hormone.

Min.: minutes; GBH: Glyphosate Group; CTRL: Control Group; AUC: area under curve.

### Liver histology in male F1 offspring

**Figure 4:**
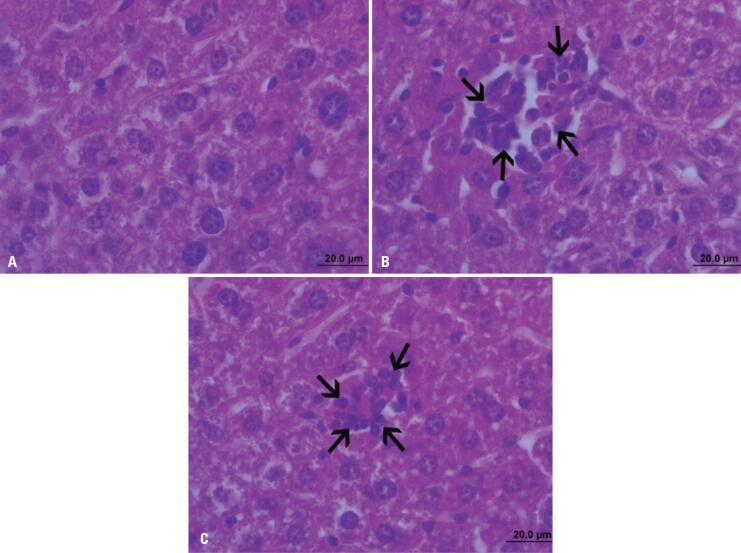
Effect of maternal exposure to glyphosate on F1 offspring liver tissue. (A) Normal liver (CTLR-F1 mice); (B and C) Leukocytic infiltrate (GBH-F1 mice). Hematoxylin and eosin staining Scale bar, 20.0*μ*m.

Mice in the CTRL-F1 and the GBH-F1 Group had normal hepatic tissue, with no fat accumulation in hepatocytes or excessive connective tissue deposition. Mice in the CTRL-F1 Group had no inflammatory foci. Hepatocellular ballooning was not observed in any of the F1 groups (Table 3; Figure 4A). However, mild to moderate lobular inflammation characterized by leukocytic infiltrates in hepatic tissues was observed in 60% and 20% of GBH-F1 mice respectively (Table 3; Figures 4B and C).

**Table 3:** Histological features of Control-F1 and Glyphosate-F1 mice on postnatal day 150

Histological feature	Definition	% in each category, according to group		
		Score/code	CTRL-F1 n=5 (%)	GBH-F1 n=5 (%)
Steatosis: Grade	Low to medium power evaluation of parenchymal involvement by steatosis			
	<5%	0	100	100
	5%-33%	1	0	0
	>33%-66%	2	0	0
	>66%	3	0	0
Location	Predominant distribution			
	Zone 3	0	0	0
	Zone 1	1	0	0
	Azonal	2	0	0
	Paracinar	3	0	0
Microvesicular steatosis	Continuous grouping			
	Absent	0	100	100
	Present	1	0	0
Fibrosis: stage	None	0	100	100
	Perisinusoidal or periportal	1	0	0
	Middle, zone 3, perisinusoidal	1A	0	0
	Moderate, zone 3, perisinusoidal	1B	0	0
	Portal/periportal	1C	0	0
	Perisinusoidal and portal/periportal	2	0	0
	Bridging fibrosis	3	0	0
	Cirrhosis	4	0	0
Inflammation: lobular inflammation	Overall assessment of inflammatory foci			
	No foci	0	100	20
	<2 foci per 200X field	1	0	60
	2-4 foci per 200X field	2	0	20
	>4 foci per 200X field	3	0	0
Liver cell injury: ballooning	None	0	100	100
	Few balloon cells	1	0	0
	Many cells/prominent ballooning	2	0	0

CTRL: Control Group; GBH: Glyphosate Group.

## DISCUSSION

Exposure of C57Bl/6 female mice to glyphosate (Roundup Original DI^®^) during pregnancy and lactation resulted in lower body weight gain (day 21 and day 30, respectively). As previously shown, duration of gestation and number of pups did not differ between maternal groups.^(20)^ According to Ait Bali et al.^(23)^ reduction in body weight is an important indicator of toxicity and may reflect the ability of glyphosate to produce reactive oxygen species. The fact that female mice exposed to glyphosate had lower water and food intake should be emphasized. Lower water and food intake may have contributed to lower body weight gain during the experimental period in this study. Beuret et al.^(17)^ and Daruich et al.^(19)^ reported lower body weight and/or water and food intake in pregnant rats exposed to glyphosate. Authors of those studies suggested this reduction may be due to the poor palatability of glyphosate or the effects of glyphosate and/or its metabolites on the thirst center in the hypothalamus. Pandey et al.^(9)^ suggested that, besides potential toxic effects, lower body weight and food intake may reflect the endocrine disrupting effect of Roundup Original DI^®^.

Exposure to glyphosate (Roundup Original DI^®^) at 0.5% may have impaired gestation initiation or complete development (19%) and induced changes in parental care (38%), accounting for 57% of mice exposed to glyphosate in this study. Milesi et al.^(24)^ reported lower numbers of implantation sites associated with higher rates of pre-implantation embryo loss in females exposed to different doses of glyphosate (2 and 200mg/kg) during the perinatal period. According to Camargo et al.^(25)^ exposure to toxic agents in the early phases of development (gametogenesis, pre-implantation and implantation) may cause embryolethality and fetal malformations. Early exposure to EDCs may affect maternal care.^(26)^ These effects may account for differences in the fetal maturation rate, including growth and subsequent neuroendocrine and behavioral responses derived from epigenetic changes.^(27)^

Exposure to EDCs during fetal development (*i.e*., during gestation and lactation) may impact fetal development and lead to permanent changes in organ and tissue function, with a higher risk of disease development in adult life. Some EDCs have also been shown to have biphasic, age-dependent effects.^(28,29)^ Given their deregulatory properties, EDCs may impact glycoregulatory hormones.^(30)^ Pre and postnatal exposure to glyphosate (Roundup Original DI^®^) increased glucose sensitivity in the GBH-F1 Group on PND60 in this study. Although this parameter was normalized by PND143, glucose level decline after insulin administration was significantly higher in the GBH-F1 compared to the CTRL-F1 Group, suggesting higher sensitivity to this hormone. Of note, fasting plasma glucose and insulin levels did not differ between the GBH-F1 and CTRL-F1 Group. Tizhe et al.^(31)^ reported a subtle (non-significant) increase in plasma glucose and insulin levels in male rats exposed to different concentrations of Bushfire^®^ (a commercial formulation of glyphosate). Veissi et al.^(30)^ observed increased glycemia and decreased insulinemia in male mice exposed to Bisphenol A (BPA) (diphenol used in plastic products, with endocrine disrupting effects). According to Bonvallot et al.^(32)^ exposure to a mixture of eight pesticides commonly found in the environment may interfere with glycemic metabolism by increasing hepatic glucose production in dams and their offspring. This effect may be due to lower levels of alanine in the plasma of mothers and in the liver and brain of offspring. Alanine is a nonessential amino acid that plays an important role in the regulation of glucose metabolism. Several studies have addressed the effects of glyphosate exposure on glucose metabolism in fish, with controversial findings. Langiano et al.^(33)^ reported an increase in fish glucose levels within 24 and 96 hours of exposure to Roundup Original DI^®^. This is a very common response in fish under stress conditions. In contrast, de Moura et al. observed plasma glucose decline in fish exposed to Roundup Original DI^®^.^(14)^

The liver is the major organ responsible for detoxification and metabolism of chemical compounds due to its ability to modify xenobiotics. Hepatic injury may occur at the beginning or during organism development and may result in several disorders.^(16)^ Studies have shown that the liver is the primary organ affected by glyphosate exposure.^(17,34)^ F1 mice born of dams exposed to glyphosate (Roundup Original DI^®^) in this study did not develop hepatic steatosis. However, mild (60%) to moderate (20%) inflammatory foci were observed in the liver of these animals. Daruich et al.^(19)^ showed that exposure to glyphosate (0.5% and 1%) induces dose-dependent changes in the activity of the enzymes isocitrate dehydrogenase, glucose-6-phosphate dehydrogenase and malate dehydrogenase in the liver, heart and brain of dams and their offspring. Beuret et al.^(17)^ reported hepatic lipoperoxidation in pregnant rats exposed to 1% glyphosate. Similar effects were observed in their offspring, which also had increased activity of the enzyme glutathione peroxidase. It has been shown that glyphosate acts on vertebrates by inhibiting enzymes in the cytochrome P450 family.^(35)^ Exposure to glyphosate may cause loss of mitochondrial transmembrane potential, leading to oxidative stress in the liver.^(36)^ Apoptosis and autophagy may also play a role in glyphosate toxicity mechanisms.^(37)^

## CONCLUSION

In summary, maternal exposure to glyphosate (Roundup Original DI^®^) during pregnancy and lactation increased glucose tolerance of offspring on postnatal day 60. In spite of glucose tolerance normalization by postnatal day 143, higher insulin sensitivity was observed. This study was also the first to demonstrate that maternal exposure to glyphosate leads to hepatic inflammation in adult F1 mice offspring.
